# Syrian hamster dermal cell immortalization is not enhanced by power line frequency electromagnetic field exposure

**DOI:** 10.1038/sj.bjc.6690704

**Published:** 1999-10

**Authors:** S C Gamble, H Wolff, J E Arrand

**Affiliations:** Department of Biology and Biochemistry, Brunel University, Uxbridge Middlesex UB8 3PH, UK; Brunel Institute for Bioengineering, Brunel University, Uxbridge Middlesex UB8 3PH, UK

**Keywords:** electromagnetic fields, immortalization, mammalian cells

## Abstract

Several epidemiological studies have suggested associations between exposure to residential power line frequency electromagnetic fields and childhood leukaemia, and between occupational exposure and adult leukaemia. A variety of in vitro studies have provided limited supporting evidence for the role of such exposures in cancer induction in the form of acknowledged cellular end points, such as enhanced mutation rate and cell proliferation, though the former is seen only with extremely high flux density exposure or with co-exposure to ionizing radiation. However, in vitro experiments on a scale large enough to detect rare cancer-initiating events, such as primary cell immortalization following residential level exposures, have not thus far been reported. In this study, large cultures of primary Syrian hamster dermal cells were continuously exposed to power line frequency electromagnetic fields of 10 100 and 1000 μT for 60 h, with and without prior exposure to a threshold (1.5 Gy), or sub-threshold (0.5 Gy), immortalizing dose of ionizing radiation. Electromagnetic field exposure alone did not immortalize these cells at a detectable frequency (≥ 1 × 10^−7^); furthermore, such exposure did not enhance the frequency of ionizing radiation-induced immortalization. © 1999 Cancer Research Campaign


*British Journal of Cancer* (1999) **81**(3), 377–380
© 1999 Cancer Research Campaign
Article no. bjoc.1999.0704

### Syrian hamster dermal cell immortalization is not enhanced by power line frequency electromagnetic field exposure

**SC Gamble[1], H Wolff[2] and JE Arrand[1]**

[1]Department of Biology and Biochemistry, Brunel University, Uxbridge Middlesex UB8 3PH, UK; [2]Brunel Institute for Bioengineering, Brunel University, Uxbridge Middlesex UB8 3PH, UK

**Summary** Several epidemiological studies have suggested associations between exposure to residential power line frequency electromagnetic fields and childhood leukaemia, and between occupational exposure and adult leukaemia. A variety of in vitro studies have provided limited supporting evidence for the role of such exposures in cancer induction in the form of acknowledged cellular end points, such as enhanced mutation rate and cell proliferation, though the former is seen only with extremely high flux density exposure or with co-exposure to ionizing radiation. However, in vitro experiments on a scale large enough to detect rare cancer-initiating events, such as primary cell immortalization following residential level exposures, have not thus far been reported. In this study, large cultures of primary Syrian hamster dermal cells were continuously exposed to power line frequency electromagnetic fields of 10 100 and 1000 μT for 60 h, with and without prior exposure to a threshold (1.5 Gy), or sub-threshold (0.5 Gy), immortalizing dose of ionizing radiation. Electromagnetic field exposure alone did not immortalize these cells at a detectable frequency ($\geq 1 \times 10^{-7}$); furthermore, such exposure did not enhance the frequency of ionizing radiation-induced immortalization. © 1999 Cancer Research Campaign

**Keywords:** electromagnetic fields; immortalization; mammalian cells

After evaluation of the combined data from many years of research, a US government panel concluded in 1998 that extremely low frequency electromagnetic fields (EMFs), such as those associated with power lines and domestic electrical appliances, are possibly carcinogenic to humans (NIEHS Working Group Report, 1998), thus heightening public concern over the safety of the supply and use of electric power. Much of the data evaluated included epidemiological studies showing a weak association between EMF exposure and childhood and some workforce cancers.

The first report of an apparent association of domestic exposure to EMFs and childhood cancers (Wertheimer and Leeper, 1979) indicated a two- to threefold increased risk of childhood leukaemias and lymphomas in homes in the vicinity of electrical wiring and transformers (the so called 'wiring configuration' classification). This association was subsequently upheld in a study by Savitz et al (1988) who supplemented wiring configuration estimates of exposure with actual measurements in some homes. However, both of these studies were small and results could conceivably be attributed to indirect effects or confounders. Results from a more representative study by Feychting and Ahlbom (1993), on a very large population of people who had lived close to power lines in Sweden, again supported the association of residential EMF exposure with childhood leukaemia. No influence of possible confounders could be identified in this study. However, the number of cancer cases was low and scepticism continued. A more recent large study by Linet et al (1997) produced a somewhat disputed negative result for association of childhood acute lymphoblastic leukaemia with residential exposure to magnetic fields (Gochfeld, 1997; Levallois and Gauvin, 1997; Neutra, 1997; Stevens, 1997; Wartenberg, 1997). In taking into account these and other studies, the NIEHS working group concluded that there is limited evidence that residential exposure to extremely low frequency magnetic fields is carcinogenic to children (NIEHS Working Group Report, 1998).

The NIEHS working group also concluded that there was limited evidence for association of occupational exposure to EMFs and adult chronic lymphocytic leukaemia. This conclusion was based on several studies, including those of Floderus et al (1993) and Feychting et al (1997), where an increase in relative risk with increasing exposure was found. There was deemed to be inadequate evidence for association of other adult cancers with EMF exposure.

However, thus far, in vivo and in vitro experiments, including those with cell transformation or immortalization end points, have neither confirmed nor refuted the epidemiological studies. Furthermore, an underlying mechanism for interaction of EMFs with biological systems remains obscure, though free radical involvement and radon daughter attraction have both been suggested (Brocklehurst and McLauchlan, 1996; Henshaw et al, 1996). Confirmation of epidemiological results with animal and cell culture studies is crucial, but results of some such studies have proved controversial with replication studies sometimes contradicting initial results (Goodman and Shirley-Henderson,

*Received 5 January 1999*
*Revised 4 May 1999*
*Accepted 4 May 1999*

*Correspondence to:* JE Arrand

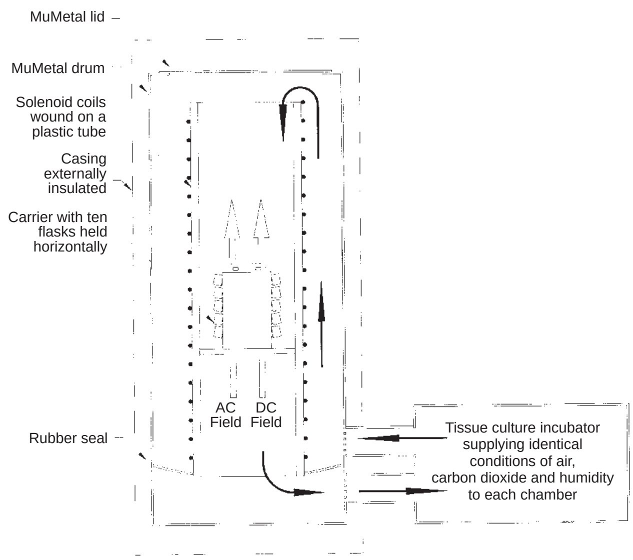

**Figure 1** A plan of one chamber of the exposure system showing the positioning of the sample flasks within the central third of the solenoid and orientations of the AC and DC fields with respect to the samples. The second chamber is identical, with an equivalent atmosphere of conditioned air, carbon dioxide and humidity being supplied by the common tissue culture incubator. Temperature is controlled by thermostats in the base of each chamber and is monitored throughout each experiment. Each chamber is surrounded by mu-metal and has a tight-fitting mu-metal lid ensuring that a field generated in one chamber does not affect the other chamber and that stray environmental fields, including earth's DC field, are excluded. DC fields equivalent to the Earth's field are generated by the same solenoids used to produce AC fields

1991; Lacy-Hulbert et al, 1995; Saffer and Thurston, 1995). Experimental problems have often been exacerbated by poor choice of target cells for some biological endpoints, poor culture conditions and controls, and experimenter bias.

Human cells are refractory to in vitro immortalization or transformation, two key, early steps in cancer progression (Newbold et al, 1982). Syrian hamster dermal (SHD) cells, however, have proved valuable as a model, low background cell immortalization system to assess the carcinogenicity of various agents, including soluble nickel which, like EMFs, does not directly damage DNA (Trott et al, 1995). This system can detect events at frequencies of less than 1 in $10^6$ over a background immortalization frequency of less than 1 in $10^9$. Experiments to detect low frequency events require exposure of large numbers of cells in a correspondingly large and uniform exposure volume. We therefore exposed SHD cells to EMFs using a large, in-house designed dual-chamber, fully shielded exposure source which allows very accurate EMF exposure under random, blinded conditions and in which effects of possible confounders (e.g. temperature and field fluctuations) can be ruled out.

Here we report on cell immortalization by EMFs, at levels commonly encountered in the home, in the presence or absence of a known carcinogen, γ-radiation.

### MATERIALS AND METHODS

### The exposure system

The exposure system was designed and constructed in-house and encompasses many features to ensure that EMF-exposed cells and mock-exposed cells remain healthy in the exposure chambers and differ only in respect of the applied field. Full details of the exposure system are to be published elsewhere (Wolff et al, 1999). However, the main design features for one of the two chambers are shown in Figure 1 and are described below.

The exposure system consists of two identical, insulated chambers in which mu-metal shielding surrounds very large solenoids wound onto perspex tubes. The central region of each solenoid delivers a very uniform field and provides ample space to house ten 75-$cm^2$ tissue culture flasks in a levelled carrier, through which both AC and DC (mock earth) fields pass perpendicular to the culture surfaces. Each chamber acts as an independent incubator, with identical conditions of conditioned air, carbon dioxide and humidity being pumped from a common tissue culture incubator. Temperature and field strength in each chamber is monitored throughout each experiment. Temperature variation between the chambers, and between any two time points within one chamber, is consistently less than 0.1°, even when the field-inducing current is running. The correspondence of the predicted magnetic flux densities with those actually generated within each chamber (as measured with an accurate fluxgate magnetometer) is within 0.9% at 1000 μT. Furthermore, the field produced in the active chamber during the course of an experiment has been shown to vary by less than 0.1% and is uniform in the central third of the length of the solenoid, which houses the sample carrier. Other common variables, such as stray fields and vibration, which could conceivably confound interpretation of biological end points, have been quantified and found to be negligible. The choice of coil to be energized is computer-regulated and decoding is carried out by an independent key card holder only after all experimental data have been analysed, thus ensuring effective blinding and excluding the possibility of experimenter bias.

As a consequence of the effective mu-metal shielding of the chambers of the exposure system, the earth's DC field is excluded. In order to rule out any effect of the absence of the earth's field, we routinely superimpose a DC field of 44 μT in both chambers (mock and exposed) throughout the experiment. In one set of experiments, however, this simulated earth's field was omitted to check the effect, if any, of its absence.

### Experimental design

The SHD cell system has previously been used successfully to measure events at frequencies of $1 \times 10^{-5}$ (Trott et al, 1995). Here, we have adapted the published procedure to incorporate tenfold more cells and thus increase the sensitivity of the assay to permit visualization of events which could occur at the extremely low frequencies suggested by human epidemiological studies. As there is effectively no dose–response or time course data for EMF effects in the literature, we chose to expose cells to three flux densities of magnitudes which might be encountered in a residential environment and for a continuous exposure time of 60 h following a 12 h equilibration period; 72 h is the maximum time for which SHD cells remain healthy and viable without subculture or feeding.

Primary SHD fibroblasts were prepared as described previously (Newbold et al, 1982) in batches of sufficient cell numbers to perform a series of carefully matched EMF exposures and sham exposures (approximately $2 \times 10^7$ cells are obtained from one newborn animal). These were cryopreserved, then thawed as required to be used at passage 3 for EMF and/or ionizing radiation exposure.

© 1999 Cancer Research Campaign

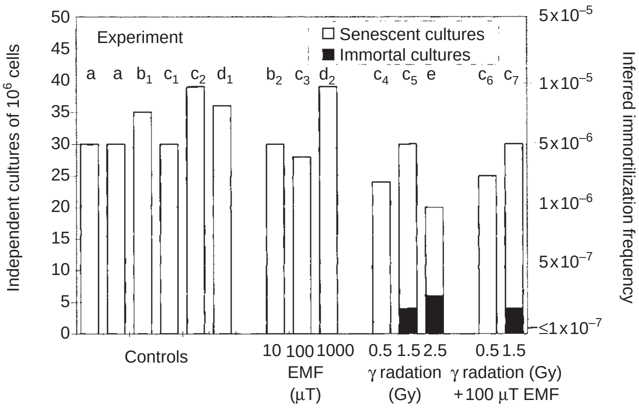

**Figure 2** Immortalization of Syrian hamster dermal cells by EMF and ionizing radiation. The vertical bars show the numbers of independent cultures which completely senesced (pale shading) or in which immortal clones arose (dark shading). The exposure conditions for duplicate experiments are detailed in Table 1. The right vertical axis shows the immortalization frequencies which can be inferred from these experiments

### Exposure of SHD cells to EMF and/or $^{60}$Co γ-rays

For exposure, ~$10^7$ passage 3 SHD cells, in Dulbecco's modified Eagle's medium (DMEM) + 15% fetal calf serum (FCS) in 5 75 $cm^2$ vented-lid tissue culture flasks ($2 \times 10^6$ per flask), were acclimatized for 12 h in each of the source chambers before random computer selection and exposure for 60 h of one chamber to 10, 100 or 1000 μT AC fields with 44 μT DC field (to mimic earth's field) applied to each chamber. All variables were continuously monitored and experiments were computer-blinded as described above. After exposure or mock exposure, each flask was assayed for immortalization. Cells were split into multiple aliquots of $1 \times 10^6$ cells which were independently subcultured at a split ratio of 1:5 until they started to senesce and then at a ratio of 1:3 (approximately seven passages; 20 population doublings in all) until senescence was complete or immortal lines had appeared. Immortalization was confirmed by subculture for up to 30 population doublings before cryopreservation.

In parallel, cell survival was measured following both exposure and mock exposure. To this end, exposed or sham-exposed cells were plated on lethally-irradiated SHD feeder layers at densities of 500 to 10 000 cells per 50-mm dish for each dose. Plates were incubated for 7 days and then stained for scoring of colonies.

For experiments to determine the additive effects (if any) of EMFs on γ-ray immortalization of SHD cells, cells were exposed, or mock exposed, to either a sub-threshold immortalizing dose of 0.5 Gy or a measurably immortalizing dose of 1.5 Gy $^{60}$Co γ-rays at a dose rate of 1.09 Gy $min^{-1}$. The EMF exposures or mock-exposures described above were then superimposed on the γ-irradiated cells. As a positive control for immortalization of the SHD cells, each cell isolate was exposed to 2.5 Gy γ-rays alone and passaged until senescence was complete and rare immortal clones had appeared.

All exposures were duplicated on two independent isolates of SHD cells. The exact exposure conditions used in each experiment are shown in Table 1. The lower case letters each indicate a full experiment, incorporating concomitant exposure and mock-exposure, and correspond to those on the bars in Figure 2.

**Table 1** Experimental exposure conditions

| | Mock exposure (controls) | EMF exposure | γ-ray exposure | γ-ray/EMF exposure |
|---|---|---|---|---|
| symbol[a] | a a $b_1$ $c_1$ $c_2$ $d_1$ | $b_2$ $c_3$ $d_2$ | $c_4$ $c_5$ e[b] | $c_6$ $c_7$ |
| EMF (μT) | 0 | 10 100 1000 | 0 0 0 | 100 100 |
| $^{60}$Co γ-ray (Gy) | 0 | 0 0 0 | 0.5 1.5 2.5 | 0.5 1.5 |

[a]Experiments labelled a, $b_{1-2}$, $c_{1-7}$, $d_{1-2}$ were concurrent; [b]positive control.

### RESULTS

The results from both independent cell isolates are summarized in Figure 2, with light shaded bars representing independent cultures in which no immortal clones were visible after complete cellular senescence; dark shaded bars (only seen in cells irradiated with above threshold $^{60}$Co γ-rays) represent cultures where rare immortal clones arose. Each bar represents an individual exposure, with bar height indicating the number of independent cultures obtained at the first passage of that culture after exposure or mock exposure.

It can be seen that no immortal clones arose from a total of ~ $1.8 \times 10^8$ cells obtained at the first passage following mock exposure (bars a, $b_1$, $c_1$, $c_2$ and $d_1$). This confirms the very low frequency of spontaneous immortalization expected in these cells.

Continuous 60 h exposure to EMF doses of 10, 100 or 1000 μT does not result in immortalization of SHD cells within our detection limits (bars $b_2$, $c_3$ and $d_2$). Furthermore, while these cells show a clear dose–response to ionizing radiation alone (1.5–2.5 Gy; experiments $c_5$ and e) over a threshold of ~0.5 Gy ($c_4$), subsequent exposure to 100 μT EMF neither lowers this threshold nor amplifies the γ-ray response ($c_6$ and $c_7$). Earth's field (or lack of it) has no effect (data not shown). Cell survival measurement showed that the EMF exposures used here did not result in any detectable cell killing (data not shown).

### DISCUSSION

A previous study by Balcer-Kubiczek et al (1996) reported that the frequency of induction of neoplastic transformation by EMFs in other rodent cell types was below their detection limit of $1 \times 10^{-3}$. The use of a much larger exposure chamber and correspondingly larger cell numbers in the experiments reported here, enabled us to substantially extend detection limits. However, even with this enhanced sensitivity, our data suggest that exposure to EMFs, at levels which might be encountered in the home, do not constitute a measurable risk of cancer initiation in normal, healthy mammalian cells. Furthermore, such exposure does not enhance the initiating potential of γ-radiation exposure.

The photon energy of a 50 Hz field is much too low to ionize, and thus damage, DNA directly. Hence, a role for low-dose EMFs in the tumour initiation step of cell immortalization might be deemed unlikely. However, there have been reports of robust biological changes which could point to a role for EMFs in multi-stage carcinogenesis. These include induction of mutation in the *HPRT* gene of mammalian cells with extremely high flux density (400 mT) EMF exposure (Miyakoshi et al, 1997). This is 400 times higher than the exposures used in the experiments reported in this paper and an order of magnitude higher than the exposures which man could conceivably expect to experience. However, a

© 1999 Cancer Research Campaign very recent study (Walleczek et al, 1999) concluded that a more realistic dose of 700 μT enhanced 2 Gy γ-ray induced mutation frequency at the same locus by 1.8-fold and that this effect was dose-dependent, being reduced at lower flux densities. EMF alone at flux densities below 1000 μT did not cause mutations. This co-mutagenic effect of EMFs and γ-rays was not reflected in cellular immortalization in the experiments reported here, despite the similarity of the doses used. However, due to X-inactivation, mutation of just one allele of the X-linked *HPRT* gene results in expression of the mutant phenotype which is thus readily detectable. If the genes controlling the switch from cellular senescence to cell immortalization are autosomal, the very rare occurrence of mutation of both alleles, or mutation of one and loss of the other, will be necessary before cellular effects are seen.

At the cellular level, while studies showing induction of specific, key oncogenes, such as *MYC*, by low EMF exposure have not been replicable (Goodman and Shirley-Henderson, 1991; Lacy-Hulbert et al, 1995; Saffer and Thurston, 1995), early genetic instability end points, such as micronucleus formation and apoptosis, have been reported in transformed (but not non-transformed) human cells following exposure to doses between 100 and 1000 μT which again suggests a role for EMFs in oncogenesis, but most likely at the level of tumour promotion (Simkó et al, 1998). EMF-enhanced cell proliferation, also a tumour promotion effect, has been reported by numerous laboratories (reviewed in Lacy-Hulbert et al, 1998). In addition, a tumour co-promotional effect of 2.45 GHz microwaves with TPA has been reported by Balcer-Kubiczek and Harrison (1991) during the neoplastic transformation of C3H/10T$_{1/2}$ cells.

It has been suggested that EMF exposure does not cause DNA damage per se but adversely affects repair of pre-existing lesions by modification of repair enzyme activity (Walleczek et al, 1999). If cells were already compromised by repair gene defects, for instance, such effects might be exaggerated. Further to this, it has been observed that lymphocytes from aged or diseased donors are more responsive to EMF exposure (Cossarizza et al, 1989). Effects are also enhanced in sub-optimally cultured cells. Therefore, while the results presented here strongly suggest that the risk of cancer initiation from exposure to EMFs in cells from normal, healthy, young, inbred animals is extremely low ($\geq 1 \times 10^{-7}$), a higher risk to genetically- or health-compromised individuals in the human population cannot be ruled out.

### ACKNOWLEDGEMENTS

This study was supported by the EMF Biological Research Trust. We thank D Trott, A Cuthbert and R Newbold for advice on the SHD immortalization assay and L Janaway for the technical drawing.

### REFERENCES

Balcer-Kubiczek EK and Harrison GH (1991) Neoplastic transformation in C3H10T$_{1/2}$ cells following exposure to 120-Hz modulated 2.45 G-Hz microwaves and phorbol ester tumour promoter. *Radiation Res* **126**: 65–72

Balcer-Kubiczek EK, Zhang X-F, Harrison GH, McCready WA, Shi Z-M, Han L-H, Abraham JM, Ampey LL, Meltzer SJ, Jacobs MC and Davis CC (1996) Rodent cell transformation and immediate early gene expression following 60-Hz magnetic field exposure. *Environ Health Perspect* **104**: 1188–1198

Brocklehurst B and McLauchlan KA (1996) Free radical mechanism for the effects of environmental electromagnetic fields on biological systems. *Int J Radiat Biol* **69**: 3–24

Cossarizza A, Monti D, Bersani F, Cantini M, Cadossi R, Saachi A and Franceschi C (1989) Extremely low frequency pulsed electromagnetic fields increase cell proliferation in lymphocytes from young and aged subjects. *Biochem Biophys Res Commun* **160**: 692–698

Feychting M and Ahlbom A (1993) Magnetic fields and cancer in children residing near Swedish high-voltage power lines. *Am J Epidemiol* **138**: 467–481

Feychting M, Forssén U and Floderus B (1997) Occupational and residential magnetic field exposure and leukemia and central nervous system tumors. *Epidemiology* **8**: 384–389

Floderus B, Persson T, Stenlund C, Wennberg A, Ost A and Knave B (1993) Occupational exposure to electromagnetic fields in relation to leukemia and brain tumors – a case control study in Sweden. *Cancer Causes Control* **4**: 465–476

Gochfeld M (1997) Correspondence: Leukemia and exposure to magnetic fields. *New Engl J Med* **337**: 1472

Goodman R and Shirley-Henderson A (1991) Transcription and translation in cells exposed to extremely low frequency electromagnetic fields. *Bioelectrochem Bioenerg* **25**: 335–355

Henshaw DL, Ross AN, Fews AP and Preece AW (1996) Enhanced deposition of radon daughter nuclei in the vicinity of power frequency electromagnetic fields. *Int J Radiat Biol* **69**: 25–38

Lacy-Hulbert A, Wilkins RC, Hesketh TR and Metcalfe JC (1995) No effect of 60 Hz electromagnetic fields on *MYC* or β-actin expression in human leukemic cells. *Radiation Res* **144**: 9–17

Lacy-Hulbert A, Metcalfe JC and Hesketh R (1998) Biological responses to electromagnetic fields. *FASEB J* **12**: 395–420

Levallois P and Gauvin D (1997) Correspondence: Leukemia and exposure to magnetic fields. *N Engl J Med* **337**: 1471

Linet MS, Hatch EE, Kleinerman RA, Robinson LL, Kaune WT, Friedman DR, Severson RK, Haines CM, Hartstock CT, Niwa S, Wacholder S and Tarone RE (1997) Residential exposure to magnetic fields and acute lymphoblastic leukemia in children. *New Eng J Med* **337**: 1–7

Miyakoshi J, Kitagawa K and Takebe H (1997) Mutation induction by high-density, 50 Hz magnetic fields in human MeWo cells exposed in the DNA synthesis phase. *Int J Radiat Biol* **71**: 75–79

Neutra RR (1997) Correspondence: Leukemia and exposure to magnetic fields. *New Eng J Med* **337**: 1473

Newbold RF, Overell RW and Connell J (1982) Induction of immortality is an early event in malignant transformation of mammalian cells by carcinogens. *Nature* **299**: 633–635

NIEHS Working group report (1998) Assessment of health effects from exposure to power-line frequency electric and magnetic fields. EMF RAPID program, NIH, PO Box 12233, MD EC-16, USA

Saffer JD and Thurston SJ (1995) Short exposures to 60 Hz magnetic fields do not alter *MYC* expression in HL60 or Daudi cells. *Radiation Res* **144**: 18–25

Savitz DA, Wachtel H, Barnes FA, John EM and Tvrdik JG (1988) Case control study of childhood cancer and exposure to 60 Hz magnetic fields. *Am J Epidemiol* **128**: 21–38

Simkó M, Kriehuber R, Weiss DG and Luben RA (1998) Effects of 50 Hz EMF exposure on micronucleus formation and apoptosis in transformed and nontransformed human cell lines. *Bioelectromagnetics* **19**: 85–91

Stevens RG (1997) Correspondence: Leukemia and exposure to magnetic fields. *New Eng J Med* **337**: 1472

Trott DA, Cuthbert AP, Overell RW, Russo I and Newbold RF (1995) Mechanisms involved in the immortalisation of mammalian cells by ionising radiation and chemical carcinogens. *Carcinogenesis* **16**: 193–204

Walleczek J, Shiu EC and Hahn GM (1999) Increase in radiation-induced *HPRT* gene mutation frequency after nonthermal exposure to nonionizing 60 Hz electromagnetic fields. *Radiation Res* **151**: 489–497

Wartenberg D (1997) Correspondence: Leukemia and exposure to magnetic fields. *New Eng J Med* **337**: 1471

Wertheimer N and Leeper E (1979) Electric wiring configurations and childhood cancer. *Am J Epidemiol* **109**: 273–284

Wolff H, Gamble G, Barkley T, Janaway L, Jowett F, Halls JAT and Arrand JE (1999) The design, construction and calibration of a carefully-controlled source for exposure of mammalian cells to extremely low frequency electromagnetic fields. *J Radiol Protect* (in press)

 © 1999 Cancer Research Campaign
